# Miscellany

**Published:** 1906-10

**Authors:** 


### MISCELLANY.

Dr. Walter D. Greene, Health Commissioner of Buffalo, has issued the following circulars:

No. I.

On or about October 1st, 1906, there will be added to the work in the laboratory of this department, the examination for the gonococcus in cases of Opthalmia Neonatorum.

An outfit, called the "Opthalmia Outfit," consisting of two glass slides, two sterilised wire loops, together with the necessary directions and blanks of record in a mailing envelope, has been devised, and physicians may obtain the same, free of expense, by sending to the office of the Department of Health.

Directions for taking the specimens (which are enclosed with each outfit) must be carefully followed to insure reliable results. After an outfit is used it must be returned to the Department of Health by mail. Examinations will be made and reports sent as soon as possible after receipt of a used outfit.

Department of Health,
September 25, 1906.

No. II.

I beg to call attention to Section 13 of Chapter XXV of the city ordinances, which reads as follows:

"It shall be the duty of any physician, immediately upon the first visit to report to the health department all cases of infectious or contagious diseases."

There seems to be an impression that tuberculosis is not included in the list of cases to be reported. We find a great disproportion between the number of deaths from tuberculosis and the number of cases reported, and by comparing the death certificates with the reports, we see who has failed in this respect.

The Buffalo Academy of Medicine recently adopted a resolution sustaining this ordinance and calling for its enforcement.

I, therefore, request every physician to kindly do his part and *promptly* report all cases coming under observation.

The penalty for a violation of this ordinance is the same as that for all others in this chapter.

Department of Health,
September 1, 1906.

---

Rhus Toxicodendron Poisoning.—Dr. E. S. McKee, Cincinnati, (*Journal Am. M. A.* Jan. 27, 1906) found the remedy par excellence to be a saturated solution of the lead acetate in the dilute alcohol of the U. S. P. The full strength of the alcohol burns too much. Persons exposed should wash their hands immediately in the dilute alcohol, the only way to thoroughly remove the poison, which according to the investigations of Pfaff (*Journal of Experimental Medicine,* Vol. ii, No. 2) is an alcohol soluble fat resembling croton oil.

